# Co‐translational folding of α‐helical proteins: structural studies of intermediate‐length variants of the λ repressor

**DOI:** 10.1002/2211-5463.12480

**Published:** 2018-06-27

**Authors:** Yuya Hanazono, Kazuki Takeda, Kunio Miki

**Affiliations:** ^1^ Department of Chemistry Graduate School of Science Kyoto University Japan; ^2^ Present address: Graduate School of Information Sciences Tohoku University Aoba‐ku, Sendai 980‐8579 Japan

**Keywords:** cotranslational folding, crystal structure, λ repressor

## Abstract

Nascent polypeptide chains fold cotranslationally, but the atomic‐level details of this process remain unknown. Here, we report crystallographic, *de novo* modeling, and spectroscopic studies of intermediate‐length variants of the λ repressor N‐terminal domain. Although the ranges of helical regions of the half‐length variant were almost identical to those of the full‐length protein, the relative orientations of these helices in the intermediate‐length variants differed. Our results suggest that cotranslational folding of the λ repressor initially forms a helical structure with a transient conformation, as in the case of a molten globule state. This conformation subsequently matures during the course of protein synthesis.

**Database:**

Structural data are available in the PDB under the accession numbers http://www.rcsb.org/pdb/search/structidSearch.do?structureId=5ZCA and http://www.rcsb.org/pdb/search/structidSearch.do?structureId=3WOA.

**Abbreviations**
CD: circular dichroism
MBP: maltose‐binding protein
OPEP: optimized potential for efficient structure prediction
TFE: 2,2,2‐trifluoroethanol

Proteins are synthesized on the ribosomes and fold into thermodynamically stable structures. The newly synthesized polypeptides can fold cotranslationally because the folding speed and folding pathways are limited by the rate of translation 1, 2, 3, 4. The codon translation rate is 20–30 amino acids per second (in *Escherichia coli*) or 2–4 amino acids per second (in a eukaryote) 5. Consequently, various intermediate states of nascent proteins can exist for a long period because the timescale of the cotranslational folding (on the order of seconds to minutes) is much longer than that of the full‐length protein folding (on the order of microseconds) 6. Therefore, the characterizations of the intermediate states of nascent proteins are important to understand the process of the cotranslational folding. Moreover, nascent proteins, which control their own translation and quality, are involved in the regulation of the life process. Therefore, the elucidation of the mechanism of the nascent protein folding is of great importance for understanding the biological systems 7, 8. However, it is difficult to elucidate the dynamics of the cotranslational folding. The folding of nascent proteins is assisted by molecular chaperones 9 and the ribosomal surface 10, 11, 12. In addition, the nascent proteins are affected by many weak protein–protein interactions because the *in vivo* conditions are highly crowded 13. A small number of reports have analyzed cotranslational folding by means of nuclear magnetic resonance 14, 15, 16, fluorescence resonance energy transfer 16, 17, 18, and computational methods 19, 20, 21.

Recently, we reported the structures of a series of WW domain N‐terminal fragments with increasing numbers of amino acids to reveal the atomic‐level details of cotranslational folding 22. Unexpectedly, the intermediate‐length fragments formed helical structures even though the full‐length protein has no helical regions. This suggests a structural change from a structure in which short‐range interactions are decisive to one in which long‐range interactions of a particular peptide length are decisive. Therefore, the nascent proteins eventually reach the native structures by adopting stable transient conformations.

Next, to reveal the atomic‐level details of the short‐range interactions of alpha‐helical proteins in nascent protein folding, we focused on the N‐terminal domain of the λ repressor. This domain has a five‐helix bundle, and the folding mechanisms of its wild‐type and numerous variants have already been investigated using various methods 23, 24, 25, 26, 27, 28, 29, 30, 31. These studies revealed that the N‐terminal domain of the λ repressor can fold in diverse ways, including by two‐state folding, downhill folding, and helical‐intermediate folding, depending on changes in the sequence, temperature, and solvent. The full‐length folding of the λ repressor N‐terminal domain is driven by the formation of a hydrophobic core with helices. However, the folding pathway of the λ repressor cannot form such a hydrophobic core in the early stage of the peptide extension. Here, we report the results of our structural studies of two intermediate‐length fragments of the λ repressor N‐terminal domain (residues 1–20: λ_1–20_; 1–45: λ_1–45_). Intermediate‐length fragments of the λ repressor adopt a helical structure in the same way as the full‐length λ repressor (λ_1–92_). However, the relative orientation of the two helices in λ_1–45_ is not identical to that of the full‐length λ repressor.

## Materials and methods

### Preparation of proteins

The genes for expression of the intermediate‐length λ repressor N‐terminal domain (λ_1–20_ or λ_1–45_) fused with MBP at its C terminus were inserted into a pET22b vector using NdeI/HindIII sites. The linker sequences, which were Gly‐Ser‐Gly for λ_1–20_ and Gly‐Ser‐Gly‐Met for λ_1–45_, were inserted between the λ repressor fragment and MBP. The fragments of the MBP and λ repressor were amplified from the pKM596 vector (Addgene plasmid 8837) 32 and artificial gene synthesis (Hokkaido System Science, Sapporo, Japan), respectively. These constructs were transformed into Rosetta2(DE3)pLysS and grown at 37 °C in LB medium containing 100 μg·mL^−1^ ampicillin and 34 μg·mL^−1^ chloramphenicol. The protein expression was induced when the OD_600_ reached 0.6 by the addition of 1 mm IPTG at 37 °C for 3 h. After cells were harvested, the pellet was resuspended in 50 mm Tris/HCl pH 7.5 and 150 mm NaCl (Buffer A) and disrupted by sonication. The suspension of disrupted cells was centrifuged at 40 000 ***g*** for 30 min at 4 °C. The supernatant was applied to an MBPTrap column (GE Healthcare, Little Chalfont, UK) equilibrated with Buffer A. The bounded protein was eluted with Buffer A containing 10 mm maltose. Then, the pooled sample was applied to a HiLoad 16/60 Superdex 200 column (GE Healthcare) equilibrated with Buffer A.

The peptide of λ_1–20_ was synthesized by the Fmoc solid‐phase method and purified to > 95% by GL Biochem Ltd (Shanghai, China). The peptides of λ_1–45_ and λ_1–92_ were subcloned into the pET22 vector using NdeI/HindIII sites. These were fused with MBP at the N‐terminal and linked with a Gly‐Ser‐Gly‐Ile‐Glu‐Gly‐Arg linker, which contained a factor Xa recognition sequence. These constructs were expressed and purified as described above. After the gel filtration, these samples were cleaved with factor Xa (Novagen, Madison, WI, USA) in a solution containing 50 mm Tris/HCl pH 8.0, 100 mm NaCl, and 5 mm CaCl_2_ for 16 h at 20 °C. The cleaved fragments were separated by a Superdex 75 10/300 column (GE Healthcare) equilibrated with Buffer A.

### Crystallographic analysis

Two intermediate‐length λ repressor N‐terminal domains (λ_1–20_, λ_1–45_) fused with MBP (λ_1–20_‐MBP, λ_1–45_‐MBP) were concentrated to 20 mg·mL^−1^ in 10 mm Tris/HCl pH 7.5, 150 mm NaCl, and 10 mm maltose. The crystals of λ_1–20_‐MBP and λ_1–45_‐MBP were grown in a solution made up of a 1 : 1 mixture of the protein solution and reservoir solution. The reservoir conditions differed for the individual variants as follows: 1.6 m triammonium citrate was used for λ_1–20_‐MBP, and 1.6 m dl‐malic acid (pH 7.0) was used for λ_1–45_‐MBP. The X‐ray diffraction intensities were collected at BL41XU of SPring‐8 (Harima, Japan). Diffraction data sets were processed and scaled using the hkl2000 software package 33. The structure was solved by the molecular replacement method with the program molrep 34 in the ccp4 software suite 35. MBP (PDBID: http://www.rcsb.org/pdb/search/structidSearch.do?structureId=1ANF) 36 was used as a search model. The program phenix.autobuild 37 was employed for autotracing. The output structure was manually improved with the program coot 38. The structure was refined using the program phenix.refine 37. The refined structure was validated with the program molprobity 39. The crystallographic and refinement statistics are listed in Table 1. The superimpositions were performed with the program lsqkab 40. All figures for the molecular models were prepared using the program pymol 41. Coordinates and structure factors of λ_1–20_‐MBP and λ_1–45_‐MBP have been deposited in the Protein Data Bank under the accession numbers http://www.rcsb.org/pdb/search/structidSearch.do?structureId=5ZCA and http://www.rcsb.org/pdb/search/structidSearch.do?structureId=3WOA, respectively.

**Table 1:** Crystallographic and refinement statistics

	λ_1–20_‐MBP	λ_1–45_‐MBP
Crystal data		
Space group	*P*2_1_2_1_2_1_	*P*2_1_2_1_2
Cell parameters		
*a* (Å)	49.0	150.9
*b* (Å)	58.3	52.6
*c* (Å)	124.6	58.3
Resolution range (Å)	50–1.80 (1.83–1.80)	50–2.00 (2.03–2.00)
Reflections (total/unique)	214 992/33 855	218 770/32 189
Redundancy	6.4 (6.2)	6.8 (6.2)
Completeness (%)	100 (100)	99.9 (99.6)
*I/*σ*(I)*	17.5 (1.6)	15.1 (11.3)
*R*_sym_ a (%)	8.3 (81.7)	12.6 (17.9)
Refinement		
No. of atoms	3467	3727
Protein atoms	3071	3205
Ligand/Ion	36	23
Water molecules	360	499
Average B‐factor (Å^2^)	29.6	18.2
Protein atoms	28.7	17.0
λ repressor/linker/MBP	83.3/61.7/26.5	27.1/53.7/15.5
Maltose	22.5	10.7
Water molecules	37.3	26.3
*R*_work_ b */R*_free_ c (%)	17.3/21.5	15.8/19.4
RMSD bonds (Å)/angle (°)	0.006/1.0	0.009/1.1
Ramachandran plot (%)^d^	97.7/2.3/0	98.8/1.2/0

Values in parentheses refer to the highest resolution shell.

a *R*_sym_ = Σ_hkl_Σ_i_| *I*_hkl,i_−<*I*_hkl_>|/Σ_hkl_Σ_i_ *I*_hkl,i_.

b *R*_work_ = Σ_hkl_||*F*_obs_|−|*F*_calc_||/Σ_hkl_|*F*_obs_|.

c *R*_free_ was calculated using the 5% of the reflections that were not included in the refinement as a test set.

d Favored/allowed/outliers.

### *De novo* modeling

Conformational modeling of the intermediate‐length λ repressor N‐terminal domain was performed by the program PEP‐FOLD (web server http://bioserv.rpbs.univ-paris-diderot.fr/services/PEP-FOLD3/) 42. Energy evaluation by PEP‐FOLD depends on the optimized potential for efficient structure prediction (OPEP) coarse‐grained force field.

### Circular dichroism spectroscopy

Two intermediate‐length proteins (λ_1–20_ and λ_1–45_) and a full‐length (λ_1–92_) protein were separately dissolved in 5 mm potassium phosphate buffer (pH 7.5) in the presence of 0–50% 2,2,2‐trifluoroethanol (TFE). All samples were measured using a J‐805 CD spectropolarimeter (Jasco, Tokyo, Japan) in a range from 190 to 250 nm with a 1‐mm quartz cuvette. The secondary structure content was analyzed with the program JWSSE‐408 (Jasco) using a reference data set 43. The thermal transition curves of the half‐length proteins and a full‐length protein were measured with an ellipticity at 222 nm in a range from 5 °C to 85 °C.

## Results

### Crystal structure of the intermediate‐length λ repressor N‐terminal domain

**Figure 1:**
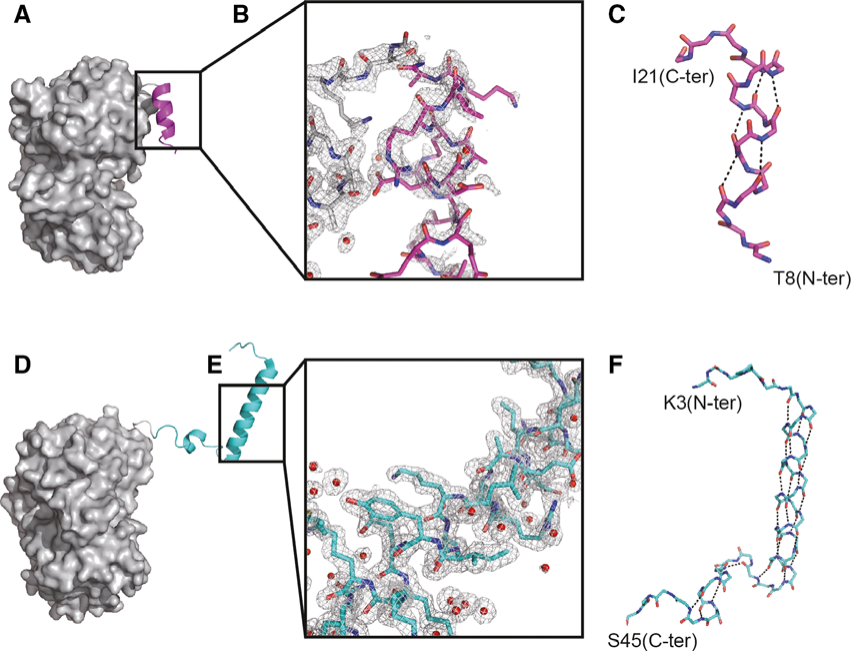
Crystal structure of the intermediate‐length λ repressor N‐terminal domain fused with MBP. (A) Overall structure of the λ_1–20_‐MBP. The regions of the λ repressor and linker are shown as a ribbon model, and the MBP is shown as a surface model. The λ_1–20_ and linker region are colored magenta and gray, respectively. (B) A 2*F*o–*F*c map for the λ_1–20_‐MBP is represented as a gray mesh contoured at the 0.8σ level. Water molecules are shown as red spheres. (C) Backbone diagram of the λ_1–20_ shown as a stick model. Hydrogen bonds are shown as black dashed lines. (D) Overall structure of the λ_1–45_‐MBP. The λ_1–45_ and linker region are colored cyan and gray, respectively. (E) A 2*F*o‐*F*c map of the λ repressor regions for the λ_1–45_‐MBP contoured at the 1.0σ level. (F) Backbone diagram of the λ_1–45_ shown as a stick model.

**Figure 2:**
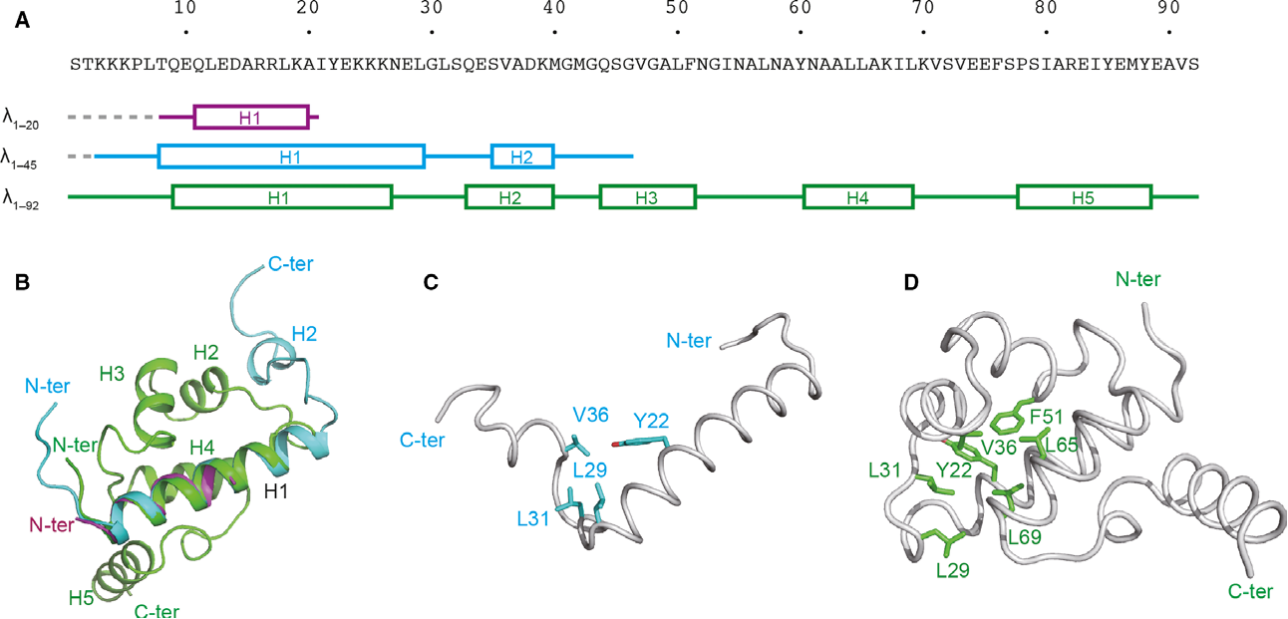
Structural difference between the intermediate‐length and full‐length λ repressor. (A) Regions of the secondary structure of the λ_1–20_, λ_1–45_, and λ_1–92_. The cylinders indicate alpha‐helices. (B) Superimposition of the λ_1–20_ (magenta), λ_1–45_ (cyan), and λ_1–92_ (green). These proteins are superimposed in the region of helix 1 (residues 9–26). (C) Hydrophobic interaction between helices 1 and 2 of the λ_1–45_. (D) Hydrophobic core of the λ_1–92_.

We determined the two crystal structures of the intermediate‐length λ repressor N‐terminal domain (λ_1–20_‐MBP and λ_1–45_‐MBP) at 1.8 Å and 2.0 Å, respectively (Fig. 1 and Table 1). To fix the C terminus so that it corresponded with that in the protein synthesis on the ribosome, MBP was fused just behind the N‐terminal fragments. As in the case of the full‐length protein, the intermediate‐length variants were made of alpha‐helices. There was little interaction between the regions of the λ repressor and MBP in the crystal (Fig. 1A,D). In addition, MBP was not an obstacle for the helix–helix interaction in the crystal of λ_1–45_‐MBP. The helical region of λ_1–20_, which contains a portion of helix 1 of the full‐length λ repressor, was almost the same as that of the full‐length protein. λ_1–20_ and the full‐length proteins (PDBID: http://www.rcsb.org/pdb/search/structidSearch.do?structureId=1LMB) could be superimposed on each other with a root‐mean‐square deviation (RMSD) of 0.77 Å. λ_1–45_ also formed a helical conformation, whose regions are almost identical to those of the full‐length protein. However, helix 2 of λ_1–45_ was slightly shorter than helix 2 of the full‐length protein (Fig. 2A). In addition, the region of residues 40–45, which are a part of helix 3, did not form any helical structures. The λ_1–45_ and full‐length proteins could be superimposed on each other with an RMSD of 3.59 Å for the region of residues 10–39 between helices 1 and 2 (Fig. 2B). On the other hand, the RMSD for the region of residues 40–45 was 9.81 Å. Moreover, the interaction between helices 1 and 2 of λ_1–45_ was different from that of the full‐length protein. The helix–helix interaction in λ_1–45_ consisted mainly of a hydrophobic interaction between Y22, L29, L31, and V36 (Fig. 2C). In the case of the full‐length protein, the relative disposition of helices 1 and 2 was dictated by the hydrophobic core, which was constructed of Y22, L29, L31, V36, F51, L65, and L69 (Fig. 2D). Although the interaction between helices 1 and 2 of the half‐length variant was similar to that of the full‐length protein, the interactions between the side chains of the half‐length and full‐length proteins, which contained Y22, L29, L31, and V36, were different. It is thus possible that the relative orientations between helices change during synthesis of the nascent polypeptide on the ribosome.

### *De novo* modeling of the intermediate‐length λ repressor N‐terminal domain

**Figure 3:**
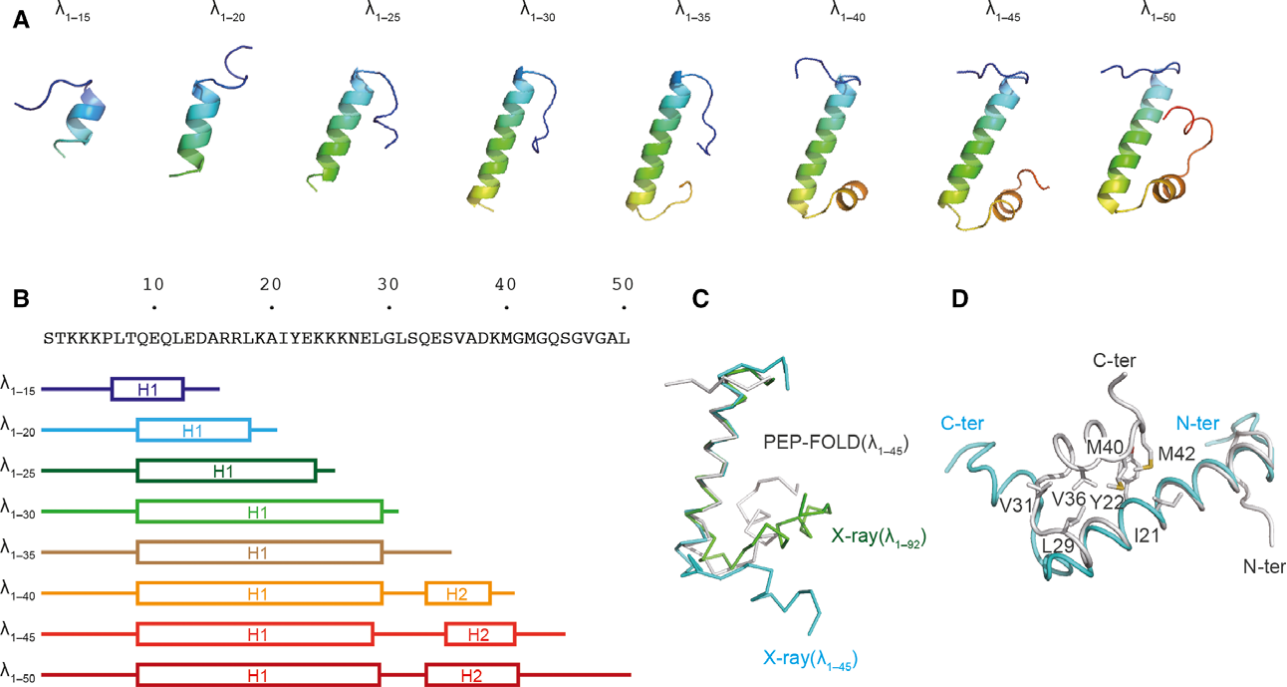
Conformational modeling by the PEP‐FOLD program. (A) The most representative of the best models for each of the intermediate‐length λ repressors. (B) Secondary structure of the best models. (C) Comparison between the conformational model (gray) and the crystal structures (λ_1–45_: cyan; and λ_1–92_: green). (D) Hydrophobic interaction between helices 1 and 2 of the conformational model of λ_1–45_ with the best score. The conformational model and the crystal structures are shown in gray and cyan, respectively.

To reveal the structural change of the λ repressor fragments as their amino acid length increased, we carried out conformational modeling with the OPEP coarse‐grained force field. First, we performed the conformational modeling of λ_1–20_ and λ_1–45_ (Figs 3A and S1). The structures of λ_1–20_ and λ_1–45_ harbored a helical conformation. In addition, the regions of helical conformation were approximately the same as those in the crystal structures (Fig. 3B). The conformational modeling yielded results similar to those of the X‐ray crystallography. Therefore, a *de novo* approach by this method can provide a reliable structure. Subsequently, we performed the conformational modeling of λ_1–15_, λ_1–25_, λ_1–30_, λ_1–35_, λ_1–40_, and λ_1–50_. All of the predicted structures of the intermediate‐length λ repressor harbored helical conformations in the same range as λ_1–20_ or λ_1–45_. This result suggested that the intermediate‐length fragments of the λ repressor were able to form a stable helical conformation. The relative orientation of the conformational model between the helices 1 and 2 was different from the orientation of the crystal structures (Fig. 3C). This orientation of the predicted structure of λ_1–45_ with the best score was mainly dictated by the hydrophobic interaction between I21, Y22, L29, V31, V36, M40, and M42 (Fig. 3D). The interaction among Y22, V36, and M40 was well observed in the predicted models (Fig. S2), whereas Y22 interacted with V36 and without M40 in the crystal structure (Fig. 2C). In the case of λ_1–92_, Y22 interacted with V36 and F51 (Fig. 2D). This result suggests that the orientation of λ_1–45_ between the helices 1 and 2 was produced by the weak and transient interaction via the native conformation.

### CD spectroscopy analysis

**Figure 4:**
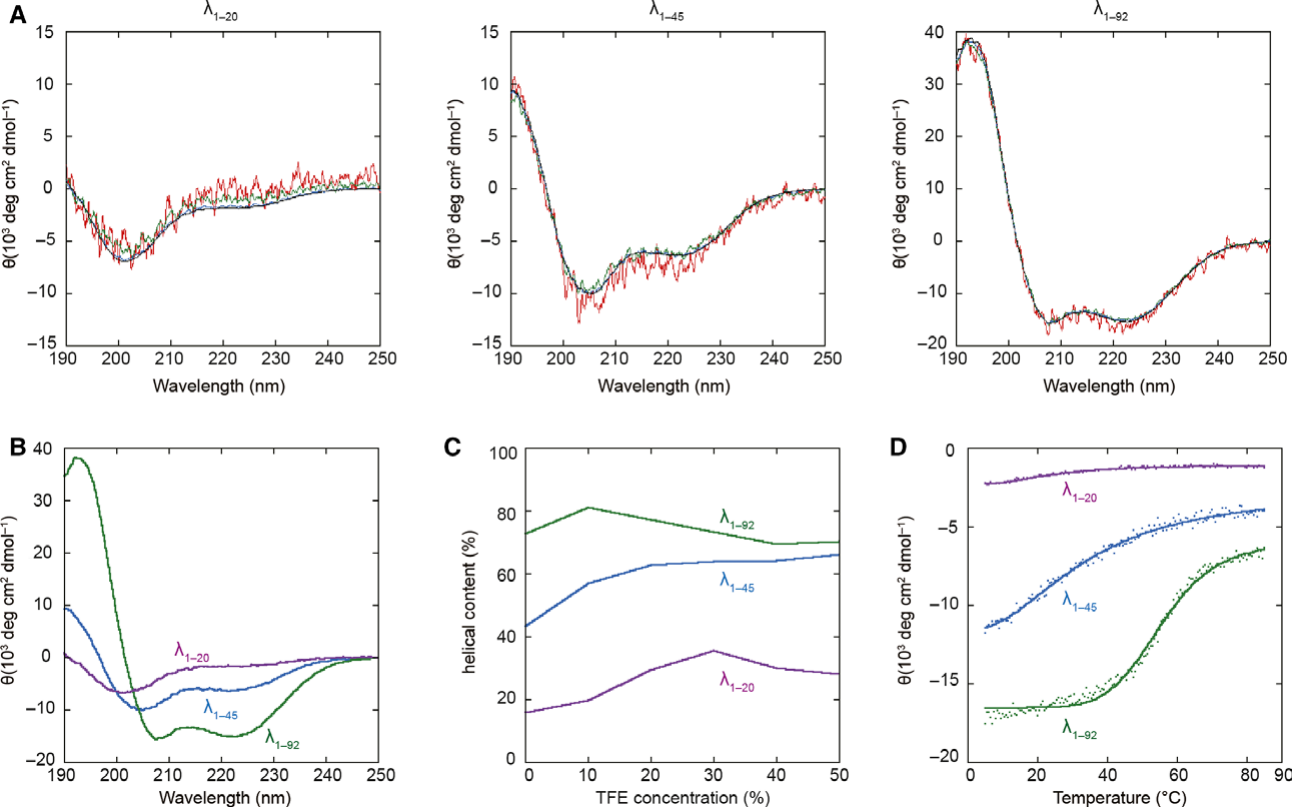
Circular dichroism spectroscopic studies comparing the intermediate‐length and full‐length λ repressor. (A) CD spectrum of the λ repressor at different concentrations. (A) CD spectrum of the λ_1–20_, λ_1–45_, and λ_1–92_ of 0.01 mg·mL^−1^ (red), 0.03 mg·mL^−1^ (green), 0.1 mg·mL^−1^ (blue), and 0.3 mg·mL^−1^ (black). (B) Comparison of the CD spectra of the λ_1–20_ (magenta), λ_1–45_ (cyan), and λ_1–92_ (green) in 0.1 mg·mL^−1^. (C) Change of the helical content as a function of TFE concentration. The λ_1–20_, λ_1–45_, and λ_1–92_ are colored magenta, cyan, and green, respectively. (D) Thermal denaturation of the λ_1–20_ (magenta), λ_1–45_ (cyan), and λ_1–92_ (green) obtained from the ellipticity at 222 nm. These data are fitted by sigmoidal curves.

The circular dichroism (CD) spectra for λ_1–20_, λ_1–45_, and λ_1–92_ were independent of the protein concentration between 0.01 and 0.3 mg·mL^−1^ (Fig. 4A). This indicates that the concentration of the proteins had little effect on the secondary structures in solution. The negative ellipticities of λ_1–20_ and λ_1–45_ at 222 nm were smaller than that of λ_1–92_ (Fig. 4B). We next analyzed the secondary structure of λ_1–20_, λ_1–45_, and λ_1–92_ from the CD spectrum. λ_1–20_, λ_1–45_, and λ_1–92_ contained 16%, 43%, and 73% helical structures in aqueous solution (Fig. 4C). The TFE titration results indicate that all of the λ repressor fragments showed a similar propensity to form a helical conformation, regardless of the concentration of TFE. In the presence of a 30% TFE concentration whose dielectric constant is close to the *in vivo* condition 44, 45, λ_1–20_, λ_1–45_, and λ_1–92_ contained 36%, 64%, and 73% helical structures, respectively (Fig. 4C). These values were in good accord with the crystallographic results, which gave helical contents of 20%, 52%, and 67%, respectively. Thermal stability was assessed by following changes in the spectrum at 222 nm with increasing temperature. The decreases in negative ellipticity of the intermediate‐length variants were approximately linear, whereas that of λ_1–92_ was sigmoidal (Fig. 4D). The melting points of the intermediate‐length variants were obscure, whereas the melting point of λ_1–92_ was clearly defined at 55 °C. It has been reported that the thermal denaturation transitions of the short peptides that have no hydrophobic core tend to be more linear than those of the globular proteins 46.

## Discussion

Our crystallographic results showed that the λ repressor of N‐terminal fragments could fold into helical structures with lengths of 20 and 45 residues. The helical regions of the fragments were in accord with the full‐length λ repressor. A *de novo* approach produced similar results. CD spectrum analysis indicated that the major part of the intermediate‐length λ repressor retained a substantial portion of helices. A previous report established that the nascent proteins exist under highly crowded conditions when the polypeptides are synthesized 47. Further, it is known that dielectric constant is significantly reduced under such crowded conditions 48. The results of our TFE titration using the CD spectrum showed that the intermediate‐length fragments folded into a helical structure even in the absence of TFE. These results support the notion that the crystal structures of the intermediate‐length fragments were well representative of diverse conditions. The various relative orientations between helices 1 and 2 were observed in the crystal and predicted structures. This indicates that the relative orientation is dictated by accidental weak hydrophobic interaction. The conformation of λ_1–45_ in crystal was shown to be a suitable model of the transient structures in peptide extension.

Based on the crystallographic, computational, and spectroscopic studies, the helical conformation is formed at the early stage of protein synthesis on the ribosomes. A theoretical analysis reported that the newly synthesized polypeptides are prone to forming a helical conformation in the ribosomal tunnel 49. In fact, alpha‐helical or helical‐like structures have been observed within the tunnel by cryo‐electron microscopy experiments 50, 51. On the other hand, the relative orientation between the helices is different from the native structure. In general, globular proteins have tightly packed hydrophobic cores and the hydrophobic effect is the major driving force behind their folding 52, 53. The λ repressor N‐terminal domain also has hydrophobic cores, and the cores are important for protein stability 54. However, the hydrophobic core matures in a later phase of the cotranslational folding. Therefore, the most stable conformation for the length is dominant in an early stage of the cotranslational folding. The transient conformation in full‐length folding is known as a molten globule state, which has a native‐like secondary structure but without a tightly packed conformation 55. Based on the results of our CD spectroscopic analysis, the fragments of the intermediate‐length λ repressor form a helical conformation but not a stable tertiary structure.

**Figure 5:**
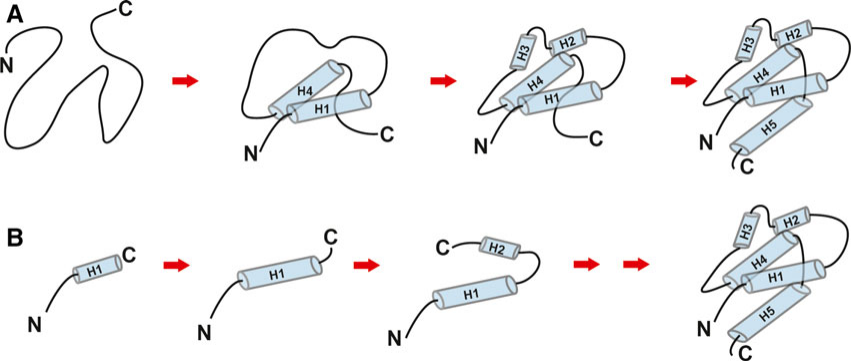
Schematic model of the folding pathway of the λ repressor. (A) The folding pathway of the full‐length protein. Cylinders indicate alpha‐helices. (B) The cotranslational folding pathway.

The folding path of the full‐length λ repressor N‐terminal domain suggests that helices 1 and 4 mainly come into contact in the earlier stages based on molecular dynamics 27, 56, 57 and mutational analysis 58. Later, helices 2 and 3 are organized to form a helical conformation, and helix 5 finally folds (Fig. 5A). However, this folding path is only possible in the case of the full‐length protein. The nascent protein could be predisposed to fold hierarchically because its folding depends on the rate of ribosome biosynthesis in the living cells, and thus, the nascent protein takes much more time to finish its folding than the full‐length protein folding. In cotranslational folding, the region of helix 1 may adopt a helical conformation and helix 2 may subsequently adopt an orientation with the most stable conformation for each length (Fig. 5B).

In this study, we revealed that the intermediate‐length λ repressor N‐terminal domain forms an alpha‐helical structure, the secondary structures of which are almost the same as the full‐length structures. However, the interaction between helices 1 and 2 of the intermediate‐length variant is different from the same interhelix interaction in the full‐length protein, because the half‐length variant has no hydrophobic cores. Formation of the λ repressor initially takes place via the local interaction between helices 1 and 2. A complete picture of this phenomenon cannot be derived merely from a folding investigation of the full‐length protein. Therefore, the present results will contribute to elucidation of the process of cotranslational folding.

## Author contributions

YH performed the biochemical and crystallographic experiments. YH, KT, and KM discussed the results and wrote the manuscript.

## Supporting information


**Fig. S1.** The most representative of the five best models for each of the intermediate‐length λ repressors.
**Fig. S2.** Hydrophobic interaction between helices 1 and 2 of the conformational models of λ_1–45_.
