# Supplementary material for: Co‐translational folding of α‐helical proteins: structural studies of intermediate‐length variants of the λ repressor
Source: FEBS Open Bio. 2018 Jun 27;8(8):1312–21. doi: 10.1002/2211-5463.12480 (PMC6070647; doi:10.1002/2211-5463.12480)
Supplement: Supplementary file 1 — Fig. S1. The most representative of the five best models for each of the intermediate‐length λ repressors. Fig. S2. Hydrophobic interaction between helices 1 and 2 of the conformational models of λ1–45. [file FEB4-8-1312-s001.docx]

Supplemental information


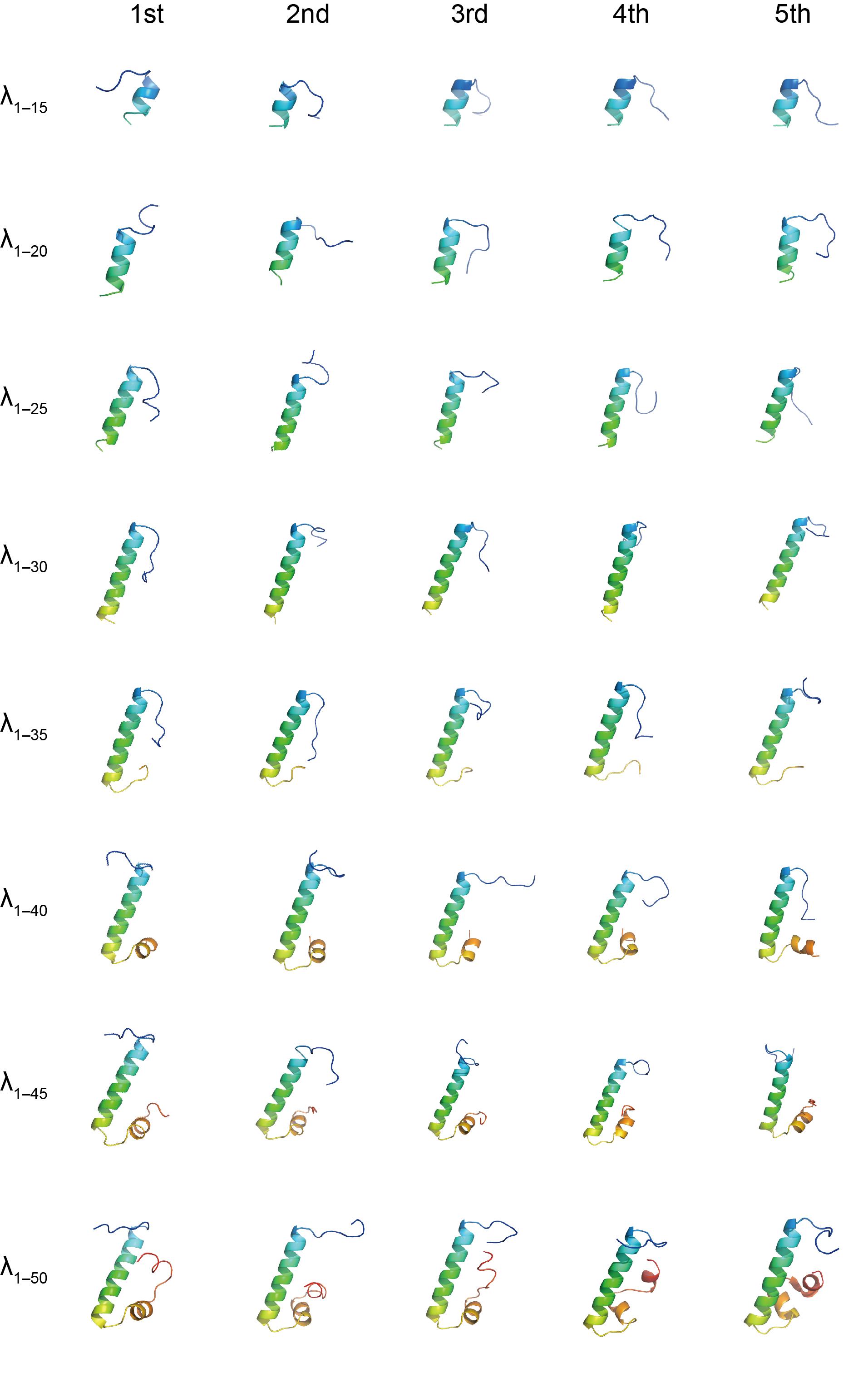


**Supplementary Fig. 1.** The most representative of the five best models for each of the intermediate-length λ repressors.

**
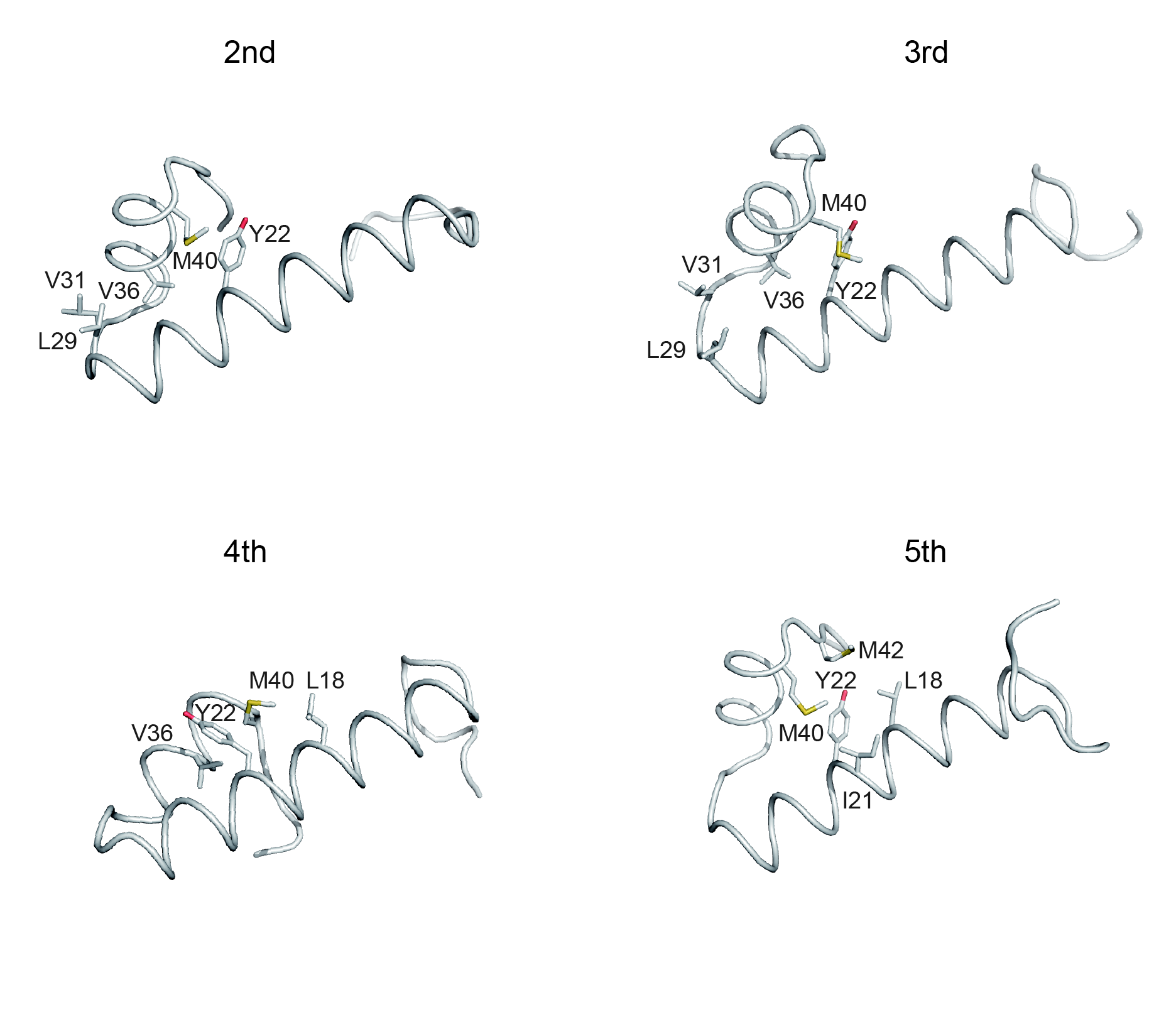
Supplementary Fig. 2.** Hydrophobic interaction between helices 1 and 2 of the conformational models of λ_1-45_.
